# Catalytic Amyloids as Novel Synthetic Hydrolases

**DOI:** 10.3390/ijms22179166

**Published:** 2021-08-25

**Authors:** Eva Duran-Meza, Rodrigo Diaz-Espinoza

**Affiliations:** 1 Departamento de Biología, Facultad de Ciencias, Universidad de Chile, Santiago 7800003, Chile; eva.lisadm@gmail.com; 2 Departamento de Biología, Facultad de Química y Biología, Universidad de Santiago de Chile, Santiago 9170022, Chile

**Keywords:** amyloids, peptides, esterase, phosphatase, di-phosphohydrolase

## Abstract

Amyloids are supramolecular assemblies composed of polypeptides stabilized by an intermolecular beta-sheet core. These misfolded conformations have been traditionally associated with pathological conditions such as Alzheimer’s and Parkinson´s diseases. However, this classical paradigm has changed in the last decade since the discovery that the amyloid state represents a universal alternative fold accessible to virtually any polypeptide chain. Moreover, recent findings have demonstrated that the amyloid fold can serve as catalytic scaffolds, creating new opportunities for the design of novel active bionanomaterials. Here, we review the latest advances in this area, with particular emphasis on the design and development of catalytic amyloids that exhibit hydrolytic activities. To date, three different types of activities have been demonstrated: esterase, phosphoesterase and di-phosphohydrolase. These artificial hydrolases emerge upon the self-assembly of small peptides into amyloids, giving rise to catalytically active surfaces. The highly stable nature of the amyloid fold can provide an attractive alternative for the design of future synthetic hydrolases with diverse applications in the industry, such as the in situ decontamination of xenobiotics.

## 1. The Amyloid State of Proteins and Peptides

**Figure 1:**
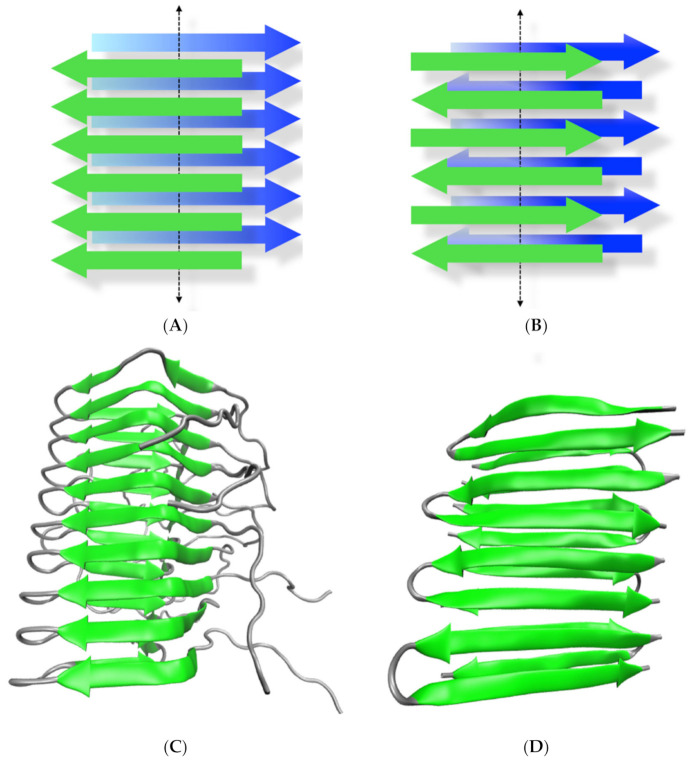
The amyloid conformation. (**A**,**B**) represent the most common beta-sheet tridimensional arrangements of amyloids in parallel and anti-parallel conformation, respectively. In each state, two intermolecular beta-sheets (depicted in green and blue) run in a cross fashion (cross beta) through the perpendicular axis of the growing fibril (dotted arrow). (**C**) Ribbon representation of the tridimensional structure of the HET protein in its amyloid conformation solved by solid-state NMR in a parallel arrangement (PDB code 2RNM). (**D**) Ribbon representation of the tridimensional structure of a de novo designed peptide in an antiparallel amyloid conformation solved by solid-state NMR (PDB code 2N1E).

Amyloids, from the word amylum (starch in Latin), are protein aggregates characterized by a highly ordered supramolecular arrangement that is stabilized by intermolecular beta-sheets [1,2]. These abnormal protein conformations have been classically associated with neurodegenerative diseases such as Alzheimer´s (AD), Parkinson’s (PD) and Creutzfeldt-Jakob disease (CJD) [3,4]. Although the proteins involved in these diseases do not share sequence homology or structural similarities in their normally folded conformations, in the pathological condition, they all misfold and aggregate as intermolecular fibrillar arrangements, indicating a convergent misfolding pathway [1,5,6]. The overall amyloid architecture exhibits a characteristic cross beta pattern in which two interacting and overlapping beta-sheets extend in a bidirectional fashion, allowing a virtually indefinite growth from both sides when a constant supply of monomeric polypeptides is provided (Figure 1A,B). The sequence-dependent intermolecular hydrogen bonding among residues of the polypeptide backbone can give rise to different amyloid arrangements such as parallel and antiparallel beta-sheets, out-of-register assembly, etc. Moreover, secondary contacts including hydrophobic, ionic, polar and aromatic stacking interactions as well as hydrogen bonds among the side chains of different residues can further shape the final amyloid conformation, modulating, for instance, the emergence of lateral interactions among longitudinal fibrils, helical bundling, fibril twisting, etc. [1,7]. Despite the ordered conformation of amyloids, the non-covalent core interactions among the monomeric polypeptide constituents charge the resulting fibrils with an inherent fragility, giving rise to heterogeneous size distributions. This heterogeneity and the low solubility of most amyloids make them difficult to characterize structurally by regular high-resolution techniques such as X-ray diffraction and NMR [8]. Still, recent advances using solid-state NMR and cryo-electron microcopy have helped to elucidate the molecular organization of several amyloids [9,10] (Figure 1C,D). 

The amyloid fold was originally thought to be exclusively accessible by certain aggregation-prone disease-associated proteins. However, examples of amyloids not associated with disease have been steadily reported in the literature during the last two decades [11,12,13,14]. These experiments showed that the amyloid state seemed to be a more general type of a novel fold. A paradigm change in this regard came with a hallmark experiment in which the SH3 domain (a very common protein domain) from PI3, a kinase protein that had never been associated with protein misfolding was successfully converted to an amyloid state in vitro [15]. Evidence for the universality of the amyloid fold comes not only from experiments on the misfolding of canonical globular proteins but also from the in vitro aggregation of peptides, including very small peptides. In AD, a 42-residue long peptide (Aβ) is proposed to be a key player in the onset and progression of the disease [16,17]. Furthermore, small peptides with sequences extracted from Aβ also form amyloids and therefore recapitulate part of the aggregation traits of the longer parent sequence. For instance, a central region containing the hydrophobic sequence KLVFF was very early shown to be critical for Aβ amyloid formation [18,19,20]. A 7-residue peptide derived from that region (KLVFFAE) has then served as a model for exploring amyloid assembly and demonstrating that small peptides can easily achieve the amyloid state in the test tube [21,22,23]. 

Structural studies based on peptides containing sequences from different pathological amyloids showed that even such small peptides can attain a diversity of morphological arrangements in the amyloid state, including parallel and anti-parallel beta-sheets, face-to-face, face-to-back, etc. [24]. Despite this conformational diversity, the smaller sizes of these peptides preclude them from achieving more complex amyloid architectures as those shown by longer peptides or misfolded proteins. The presence of non-beta regions such as loops in the sequence of these larger polypeptides appears as critical for allowing the amyloids to reach more intricate overall folds [9,10]. 

Many works have shown that amyloids can form by using not only using sequences from pathological amyloids but also de novo designed sequences [25]. Different strategies such as intercalating hydrophobic with polar or charged residues have proven successful as general approaches for designing amyloids [25,26]. Considering the hydrophobic nature of the interactions that often stabilize the cross-beta arrangement, small peptides that contain mainly hydrophobic residues are typically assembled into amyloids under different solution conditions. These results clearly support the idea that the amyloid fold is an alternative state of peptides and proteins. Interestingly, the amino acid phenylalanine can self-assemble into amyloid-like fibrils in vitro, providing strong support for an amyloid-nature of the etiology in Phenylketonuria [27]. Though a classical beta-sheet arrangement was not demonstrated, this finding remains to date the amyloid-like fibrils with the smallest possible peptide sequence.

## 2. Catalytic Activity Emerging from Peptides Self-Assembled into Amyloids

**Figure 2:**
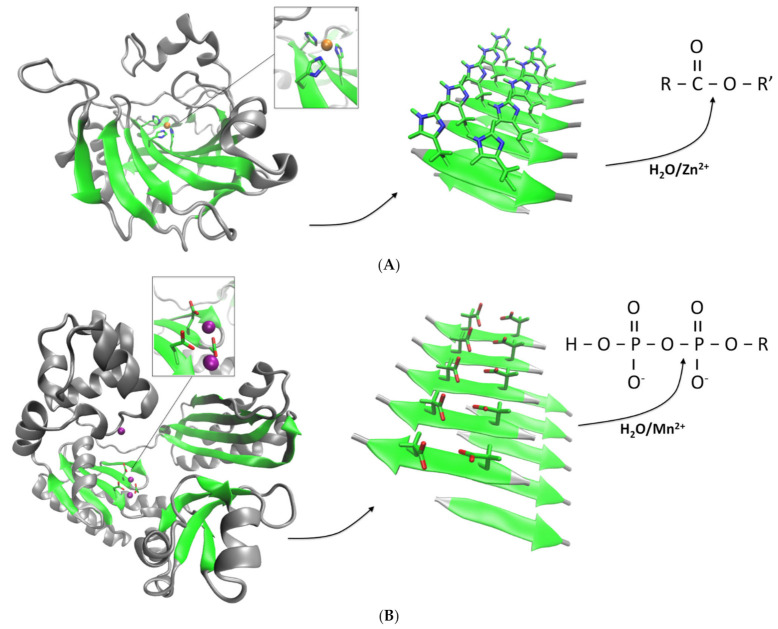
Design of catalytic amyloids with hydrolase activities. (**A**) A peptide containing histidine is designed based on the active site of CA (represented in ribbons, PDB code 1CA2) in which the catalytic histidines are located in a beta-sheet fold and coordinated with a Zn^2+^ ion (orange sphere). The peptide self-assembles into amyloids that expose the histidines to the solvent and through coordination of Zn^2+^ allows for the emergence of an esterase activity [34]. (**B**) The active sites of NTs can also be used as template for the design of catalytic amyloids. DNA polymerase IV (PDB code 1S0O) is shown in ribbons with the catalytic aspartate groups coordinated with two calcium ions (purple spheres). Peptides containing aspartate and/or glutamate form amyloids that in presence of Mn^2+^ catalyzes the specific cleavage of phosphoanhydride bonds [42,43].

The amyloid state of proteins and peptides is characterized by a very high stability compared to the unassembled monomers [18,28]. Amyloids can typically withstand in vitro high temperatures, high salinity, high pressure and pH changes [6,29]. Therefore, the self-assembled state provides the peptide or protein with emergent properties that can have applications for the design of novel bionanomaterials. Moreover, the mechanical properties of certain amyloids have revealed unique behaviors, adding to their biotechnological value [30]. Those properties emerge in part due to the beta-sheet core that stabilizes the amyloid fold. Interestingly, beta-sheets can be found to accommodate active sites alone or in combination with other secondary structures in several families of enzymes [31,32,33]. Thus, amyloids could in principle be used as a scaffold to partially mimic the catalytic activity of enzymes of interest, with the added benefit of having a high stability and unique mechanical properties. Furthermore, amyloids composed of peptides can spontaneously self-assemble, without the need of adding “activation” steps. At the molecular level, the structures of amyloids so far described have shown that hydrophobic residues typically locate in the amyloid core, whereas polar and charged residues (if present) are generally solvent-exposed. Those water-exposed residues may interact with potential substrates, charging the amyloid fold with enzyme-like reactivity, especially hydrolytic activity. Peptide sequences can then be rationally designed to create catalytically active amyloids. This concept was successfully demonstrated in the work of Rufo et al., in which the activity of carbonic of anhydrase (CA), a ubiquitous esterase, was recreated using an amyloid scaffold [34]. The active site of CA is composed of three catalytic histidine groups located in a beta-sheet, which coordinate zinc ions (Zn^2+^). Therefore, peptides that alternate histidine with hydrophobic residues can in principle coordinate Zn^2+^ while self-assembling into an amyloid state (Figure 2A). Different 7-residue sequences were thus shown to effectively form amyloids and exhibited in that state an esterase-like activity against the model compound 4-nitrophenyl acetate (pNPA), with peptide IHIHIQI forming the most active catalytic amyloid in terms of catalytic efficiency (*k_cat_*/*K_M_*). The activity was exclusively observed in presence of Zn^2+^, as it occurs with CA. The observed activities were exclusively associated to the amyloid conformation and not the monomeric species, indicating that amyloids can act as catalytically active surfaces [35]. This approach opened up new avenues for the rational design of other synthetic hydrolases.

Histidine residues are found in the active sites of many hydrolases, mainly in the form of histidine triads (including CA) [36,37]. Therefore, using histidine in the design of catalytic amyloids peptides can potentially uncover other types of hydrolytic activities. In fact, amyloids self-assembled with peptide IHIHIYI were able to catalyze the copper-dependent hydrolysis of the phosphoester bond of paraxon (phosphoesterase activity), a known organophosphate pesticide that is highly used in modern agriculture [38]. Interestingly, similar peptide sequences that previously exhibited amyloid-mediated esterase activity, including the most active sequence IHIHIQI were inactive as phosphoesterases. Only the sequences containing aromatic residues showed activity, suggesting that the activity of amyloid surfaces is sensitive to residues other than the expected catalytic groups, and rational design must be then carefully conducted. In a different work, peptide IHIHIYI also exhibited a Zn-dependent esterase activity in the amyloid state, with a catalytic efficiency even greater than peptide IHIHIQI against pNPA (Table 1) [39]. However, it is important to note that the activities of both catalytic amyloids were not measured directly in the same work and since amyloid preparations can be sensitive to the growing conditions, comparisons among catalytic activities should ideally be performed on the same experiment. 

Another group of enzymes that have their active sites directly associated with beta-sheets are the superfamily of nucleotidyltransferase (NTs). These enzymes are ubiquitous proteins that carry essential functions and are present in all kingdoms of life [31]. In general, NTs catalyze the transfer of phosphate groups between nucleotide triphosphates and a wide diversity of acceptors, and hence, they include a built-in phosphodyrolase activity. The active sites typically include one to three carboxylate groups (aspartate and/or glutamate) that coordinate magnesium or manganese (Mn^2+^) ions. In many cases, it is observed that two of these residues are located in one strand whereas a third group resides in an opposing strand. Similar to the approach used to design the esterase-like amyloids based on CA, we used the active sites of NTs as templates for developing novel catalytic amyloids (Figure 2B) [42]. However, our peptides have sequences that match exactly part of the active site sequence of a NT. The first catalytic amyloid thus designed (peptide NADFDGFQMAVHV) is based on a sequence derived from the active site of an RNA polymerase, which in turn contains one of the most conserved sequences in nature (DFDGD). A second, smaller peptide based on the active site sequence of a DNA polymerase from *Staphylothermus marinus* (peptide SDIDVFI) also yielded catalytically active amyloids [43]. Both amyloids were able to catalyze the hydrolysis of the phosphoanhydride bonds of adenosine triphosphate (ATP) in a Mn^2+^-dependent fashion. In the case of peptide SDIDVFI, the activity of the self-assembled amyloids was not restricted to ATP but affected all other ribonucleotides, and even deoxyadenosine triphosphate (dATP). Considering no other parts of the nucleotides were affected, the observed activity is consistent with the specific phosphohydrolase activity observed in NTs. 

The need of a divalent metal for the activity is not a strict requirement for developing catalytic amyloids. For instance, small peptides derived from the sequence of Aβ peptide have been used as scaffolds to develop catalytically active amyloid-like peptide assemblies. As mentioned above, peptide KLVFFAE assembles into classical amyloids with anti-parallel beta-sheets at neutral pH [23]. However, replacing glutamate with leucine (peptide KLVFFAL) gives rise to amyloid nanotubes that catalyze the retro-aldol conversion of a model compound [44]. Though not a hydrolase activity, these results demonstrate that the amyloid fold can be exploited for diverse types of activities. Still, recent work showed that condensing the lysine of peptide KLVFFAL with imidazoleacetic acid resulted in a peptide (Im- KLVFFAL) that self-assembles into esterase-like amyloids [45,46]. The esterase activity in this case is achieved in a different manner than the Zn^2+^-dependent peptides described originally [34]. First, Im- KLVFFAL forms nanotube amyloid assemblies similar as the parent KLVFFAL nanotubes, and second, the esterase activity proceeds through a covalent catalysis mechanism, in which lysine engages in transient covalent bonding with the substrate. Although this peptide requires chemical modification of its original amino acid composition to become a synthetic hydrolase, the results again support the idea that amyloids serve as useful scaffolds for exploring functionality. Interestingly, the same group showed that replacing lysine with histidine in the original, unmodified KLVFFAL peptide resulted in a peptide (HLVFFAL) that, when bound to a hemin group, assembled into nanotubes exhibiting an esterase activity that was not mediated by zinc or transient covalent intermediates [47]. Another recent example of a metal-independent amyloid hydrolase was based on a poly glutamine-containing peptide [40]. When clustered in close proximity, glutamine is known to be a highly amyloid-prone residue, which can be found in the protein sequences of several classical pathological amyloids [48]. Through hydrogen bonding networks among glutamine amide side chains, the amyloid fold can get further stabilized. Based on that, peptide HSGQQKFQFQFEQQ was shown to self-assemble into esterase-like amyloids. The activity was strictly dependent on the histidine residue and though the zinc ions were not required, the overall rates were much lower than those observed with the zinc-dependent amyloid esterases (Table 1). Interestingly, fully polar peptides with sequences alternating glutamine and tyrosine result in the formation of amyloids in vitro that are stabilized by aromatic interactions [49]. Replacing all glutamines with histidine in these small peptides charges the resulting self-assembled amyloid-like peptides with metal-independent esterase catalytic activity [50]. Given the very few reports available so far on metal-independent catalytic amyloids with hydrolase activities, it is difficult to speculate on whether these amyloids operate through a common enzyme-like mechanism. In addition, most current works do not include detailed kinetic characterizations. It appears though that the catalytic rates obtained with these amyloids will be in general lower than those obtained with their metal-dependent counterparts, as already observed with peptide HSGQQKFQFQFEQQ. It may be possible that in some of these cases, the simple addition of divalent metals such as Zn^2+^ ions into the reactions may result in an enhanced catalysis, and hence, a similar mechanism to that of metal-dependent hydrolases may be at work. Future experiments should shed light on these issues. 

The catalytic properties described in metal-dependent amyloid hydrolases seem to depend not only on the metal and the catalytic residues but also on neighboring residues [34]. These residues can affect the final amyloid conformation, the catalytic rates or the type of activity itself, though the interplay between these factors is far from clear. Furthermore, combinations of different peptides can result in synergistic interactions [51]. Peptides with the sequence LHLHLXL (in which X is replaced by different amino acids) form amyloids with diverse catalytic efficiencies that directly depend on the non-catalytic X residue. When combined, the observed catalytic rates were highly increased. Synergistic activity has also been observed with other esterase-like amyloids, indicating that this could be a more general phenomenon [29,40]. On the other hand, peptide size does not seem to pose a limit for developing amyloid-based hydrolases. A three-residue peptide assembled into amyloid-like hydrogels exhibited a metal-independent esterase activity [52]. Interestingly, this peptide was devoid of capping groups as opposed to most peptide catalytic amyloids and later work showed that capping was a modulator of the assembly and the activity [53]. As observed before with the glutamine-containing peptide, the overall catalytic parameters reached much lower values than their zinc-dependent counterparts, suggesting that the metal-dependent hydrolases can have intrinsic advantages in terms of activity. More recently, it was shown that the self-assembly of even single aromatic amino acids can also yield catalytically active amyloids [41]. Due to aromatic interactions, phenylalanine in the presence of Zn^2+^ can lead to catalytically active amyloids that also exhibit esterase activity. Though strictly not proteinaceous in nature, these amyloids showed activities that were comparable to the most active Zn-dependent amyloid esterases.

In general, though the activities reported for hydrolase-like catalytic amyloids do not necessarily correlate with those observed with bona fide enzymes, several amyloids showed kinetic parameters that appear to match the levels of activity of certain enzymes. For instance, peptide IHIHIQI showed in the amyloid state specific activities greater than CA [34]. However, caution must be taken on this type of comparison since, contrary to monomeric enzymes, self-assembled peptides act as catalytic surfaces, and hence, determining the minimal catalytic unit is not straightforward (to date, there is no established consensus on that). Such a minimal catalytic unit might even vary depending upon the peptide sequence and/or the metal used for the activity. Still, the specificity constant has emerged, similar to classical enzymology, as a common indicator of catalytic efficiency of amyloids (See Table 1). In general, it is observed that esterase-like catalytic amyloids have so far exhibited the highest values of *k_cat_*/*K_M_*, which in some cases can be comparable to typical enzymes. However, the catalytic sites on the amyloid surface may not recapitulate the whole complexity observed in the active sites of enzymes, which in addition to the catalytic groups are typically surrounded by different accessory residues at specific orientations that can affect the turnover number and/or substrate specificity. We have indeed observed that our catalytic amyloids showing di-phosphohydrolase activity do not discriminate between different nucleotide substrates [43]. As mentioned above, however, engineering the non-catalytic groups of the peptides may hold potential for overcoming these current limitations. 

From a structural point of view, the mechanisms underlying the emergence of catalytic activity in the amyloid fold are far from clear, especially considering that the field is relatively recent. On one hand, most catalytic amyloids described so far seem to follow classical enzyme kinetic behaviors. However, studies on the structure–activity relationship are scarce. It has been proposed that the emergence of catalytic activity would proceed through two independent pathways: (1) recreate part of the structural organization observed in the active sites of several enzymes, and (2) provide a surface in which these mimicked active sites are repeated in an ordered and concentrated fashion, allowing substrates and others relevant molecules to bind and react in a bidimensional space as typically observed with heterogeneous catalysts [35]. The solvent-accessible residues may possess certain flexibility to orient their side chains for proper metal coordination and/or substrate binding, thereby creating virtually identical reactive pockets extending throughout the amyloid surface. The only atomistic models available so far have been reported for catalytic amyloids exhibiting Zn-dependent esterase activity [54,55]. A combination of solid-state NMR (ss-NMR) and molecular dynamics (MD) showed that an NMR-amenable derivative of peptide IHIHIQI (peptide IHVHLQI) self-assembled into catalytically active fibrils composed of parallel beta-sheets in which the dry pocket is formed by the hydrophobic residues while the polar and charged residue form a wet surface [54]. The active site was shown to emerge from the coordination of Zn^2+^ ions with three of the four solvent-exposed histidines, creating an uninterrupted network of coordinate bonds that further stabilizes the amyloid conformation. By using a similar combination of ss-NMR and MD, Song et al. explored in a later work the conformational variations emerging in the esterase-like amyloids formed by peptides IHIHIQI, IHIHIYI and IHIHIRI [55]. The level of esterase activity achieved by each sequence was shown to be highly dependent on the specific parallel or antiparallel arrangement as well as the degree of twisting in the fibrils, with a combination of parallel and twisting resulting in the most active conformations due to a higher flexibility of the amyloid fold that would allow the active residues to better mimic the active sites of enzymes. Such structural changes appear to be exclusively controlled, at least in those cases, by the non-catalytic residues (Q, Y or R) that are also solvent-accessible, reinforcing the aforementioned importance of these accessory groups. In metal-independent catalytic amyloids, molecular dynamics simulations have shown that amyloid conformations that restrict substrate binding and/or the positioning of the active residues in the catalytic grooves can affect the catalysis of a model retro-aldol reaction [44]. In support of a surface-enhanced catalysis mechanism, works on catalytic amyloids have overall demonstrated that the reported activities are mainly, if not strictly, associated with the self-assembled state of the peptide. Such demonstrations can, for instance, be accomplished by experiments in which the observed activity remains entirely in the retentate fraction after passing the amyloids through filters with high molecular weight cutoffs [34]. Alternatively, centrifugation of the samples followed by resuspension is also shown to fully retain the activity [42,43]. The risk of contaminating enzymes in the preparations is virtually nonexistent since most peptides are either chemically produced in the laboratory or commercially acquired with very high purity. However, proper controls during the experiments, including the abovementioned experiments on amyloid enrichment, must always be provided. 

## 3. Conclusions and Perspectives

The amyloid state of proteins is nowadays recognized as an intrinsic conformation of the energy landscape of most proteins and peptides. Their unique mechanical properties and stability make amyloids an attractive alternative for the design of future bionanomaterials. Though very recent, the discovery that peptides can self-assemble into catalytic amyloids that functionally resemble many of the aspects seen in enzymes has opened up a whole new field of research that is just starting to be explored. Most of the so far reported catalytic amyloids are hydrolases, indicating that this may constitute a naturally convergent functionality that emerges from the specific properties of the amyloid architecture. The exposure of reactive groups to the solvent on these surfaces appears as a natural advantage for interactions between substrates and water molecules. On the other side, reactions involving water condensation steps may turn out unfavorable to occur on the amyloid surface unless a novel rational design approach to create dry active pockets is developed. Thus, the amyloid fold constitutes an ideal scaffold to explore novel hydrolase activities. So far, three types of hydrolase activities have been demonstrated: esterase, phosphoesterase and phosphohydrolase. Esterase activity has been so far the most exploited functionality in designing catalytic amyloids. For the design, one could envision that active sites embedded in beta-sheet folds could be the only source for mimicking enzyme-like activities on an amyloid scaffold. However, in the case of esterases, the diversity of folds that give rise to this activity is not restricted to beta-sheets [56]. A similar case is seen for enzymes belonging to the family of phosphoesterases, in which we can find that in some cases such as cyclic nucleotide phosphodiesterases the folds are mainly alpha helical [57]. These observations suggest that most hydrolase activities could in principle be accessible to mimic by an amyloid scaffold. So far, metal-dependent catalytic amyloids seem to exhibit higher catalytic rates than their metal-independent counterparts. Further mechanistic studies of this aspect and the reported influence of residues surrounding the catalytic groups will certainly help to guide the design of more efficient and novel hydrolytic catalytic amyloids. Owing to their unique properties, amyloid-based hydrolases could then become an attractive alternative for industrial applications such as decontamination of xenobiotics and other organic pollutants. 

## Figures and Tables

**Table 1:** Hydrolase activities of different types of catalytic amyloids.

Hydrolase Activity	Peptide Sequence	*k_cat_*/*K_M_* (M^−1^ s^−1^)	Refs.
Esterase	IHIHIQI-Zn	62 ^a^	[34]
	IHIHIYI-Zn	355 ^a^	[39]
	HSGQQKFQFQFEQQ	0.15 ^a^	[40]
	F-Zn	10.62 ^a^ and 76.54 ^a,d^	[41]
Phosphoesterase	IHIHIYI-Cu	2.8 × 10^−2, b^	[38]
Phosphohydrolase	NADFDGFQMAVHV-Mn^2+^	5.6 × 10^−8, c^	[42]
	SDIDVFI-Mn^2+^	6.4 × 10^−8, c^	[43]

^a^ pNPA, ^b^ paraxon, ^c^ ATP, ^d^ in deionized water.
